# Qu-Du-San-Jie decoction induces growth inhibition and vascular normalization in NF2-associated vestibular schwannoma

**DOI:** 10.3389/fphar.2022.941854

**Published:** 2022-08-19

**Authors:** Jie Lin, Shi-Wei Li, Jing Zhang, Fu-Hao Chu, Cheng-Ze Li, Zhi-Xu Bie, Han-Lu Tang, Shan Gao, Ping Li, Meng-Ting Liao, Tian-Xi Xin, Fu Zhao, Pi-Nan Liu, Xia Ding

**Affiliations:** ^1^ School of Traditional Chinese Medicine, Beijing University of Chinese Medicine, Beijing, China; ^2^ Department of Neurosurgery, Beijing Tiantan Hospital, Capital Medical University, Beijing, China; ^3^ Department of Neural Reconstruction, Beijing Key Laboratory of Central Nervous System Injury, Beijing Neurosurgical Institute, Capital Medical University, Beijing, China; ^4^ Research Center for Spleen and Stomach Diseases of Traditional Chinese Medicine, Beijing University of Chinese Medicine, Beijing, China; ^5^ Institute of Regulatory Science for Traditional Chinese Medicine, Beijing University of Chinese Medicine, Beijing, China; ^6^ School of Chinese Materia Medicine, Beijing University of Chinese Medicine, Beijing, China; ^7^ Dongzhimen Hospital, Beijing University of Chinese Medicine, Beijing, China

**Keywords:** neurofibromatosis type 2, schwannoma, Qu-Du-San-Jie decoction, antiangiogenesis, therapy

## Abstract

**Background:** Neurofibromatosis type 2 (NF2) is a rare genetic syndrome that predisposes individuals to develop bilateral vestibular schwannomas (VSs) causing a high risk of life-threatening neurological complications. Traditional treatment options for NF2-associated VS usually cause neurological damage, and to date, there are no FDA-approved pharmacotherapies for NF2. The aim of this study was to evaluate the antitumor efficacy of Qu-Du-San-Jie (QDSJ) decoction, a traditional Chinese medicine formula, on NF2-associated VS and to investigate the potential underlying mechanisms.

**Methods:** Ultra high-performance liquid chromatography-mass spectroscopy (UHPLC-MS) analysis was performed to identify the components of QDSJ and their targets. To determine the relationships between the putative targets of QDSJ and the differential genes of NF2-associated VS, the drug-disease crossover genes were screened using the UHPLC-MS data combined with our previous gene expression profiling data. The differentially expressed genes were imported into the STRING database to generate a PPI network. Differentially expressed gene targets and pathways were identified using GO and KEGG pathway enrichment analyses. The *in vitro* and *in vivo* drug efficacy of QDSJ decoction was tested using a patient-derived schwannoma cell line and a patient-derived xenograft mouse model, respectively. H&E staining, immunochemistry, and immunofluorescence staining were used to evaluate the cell proliferation and tumor vessels.

**Results:** A total of 133 compounds were identified in QDSJ decoction using UHPLC-MS analysis. Network pharmacology showed that the regulation of necroptosis, apoptosis, cell cycle, angiogenesis, adherens junction, and neuroactive ligand-receptor interaction could be associated with the efficacy of QDSJ in treating NF2-associated VS. Treatment with QDSJ induced necrotic cell death and apoptosis of schwannoma cells *in vitro* and suppressed the tumor growth *in vivo*. Histopathological analysis revealed areas of cell necrosis and enlarged tumor blood vessels in the QDSJ-treated tumors. The numbers of cells positive for Cyclin D1 and Ki-67 were significantly reduced in QDSJ-treated tumors compared to control tumors. Immunofluorescence staining of CD31 and αSMA showed a decreased number and density of tumor vessels and normalized vessel structure in QDSJ-treated tumors.

**Conclusion:** Our study demonstrates that QDSJ decoction shows significant antitumor activity against NF2-associated schwannoma and is a possible candidate for future clinical trials.

## Introduction

Neurofibromatosis type 2 (NF2) is a rare tumor predisposition syndrome characterized by the development of multiple benign tumors, including schwannoma, meningioma, and ependymoma in the central and peripheral nervous system (Evans et al., 1992). Bilateral vestibular schwannomas (VSs) are the most common lesions in NF2 affecting over 90% of NF2 patients (Asthagiri et al., 2009). Due to their unique intracranial location, bilateral VSs can cause progressive disabilities including sensorineural hearing loss and facial paralysis, and compress the brainstem, resulting in hydrocephalus, blindness, and even death (Dirks et al., 2012; Forde et al., 2021; Li et al., 2021). Traditional treatment options for NF2-associated VS include surgical resection and stereotactic radiosurgery (SRS) (Goldbrunner et al., 2020). However, surgical outcomes in terms of preservation of hearing and facial functions are much poorer in NF2 patients than in patients with sporadic VS (Samii et al., 1997; Brackmann et al., 2001; Zhao et al., 2018a). Although stereotactic radiosurgery (SRS) is potentially effective in the local control of small tumors or residual tumors in patients with NF2 (Mathieu et al., 2007; Phi et al., 2009; Shinya et al., 2019), this procedure carries some risk of delayed cranial nerve dysfunction and malignant formation (Maducdoc et al., 2015).

Until recently, there were no trial-proven medical treatments for NF2-associated VS. The majority of clinical trials for NF2-associated VSs have applied receptor tyrosine kinase inhibitors including nilotinib, sorafenib, everolimus, lapatinib, and erlotinib, and showed mixed response rates (Plotkin et al., 2010; Ammoun et al., 2011; Karajannis et al., 2012; Goutagny et al., 2015). Recent clinical experiences have shown that treatment with bevacizumab resulted in tumor shrinkage and hearing improvement in a subset of patients with NF2 (Plotkin et al., 2009; Mautner et al., 2010; Fujii et al., 2020). Moreover, several problems including frequent parenteral administration, side effects, and re-exacerbation after discontinuation, may limit the clinical utility of bevacizumab treatment (Slusarz et al., 2014; Morris et al., 2017; Tamura et al., 2017). Thus, novel treatment approaches are still needed for NF2-associated VS.

Traditional Chinese medicine (TCM) has shown advantages in suppressing tumor progression, relieving surgical complications, increasing the sensitivity of tumors to chemo- and radiotherapeutics, and improving the survival time and quality of life of patients with tumors. (Qi et al., 2015; Wang et al., 2020; Wang et al., 2021). In this study, we used Qu-Du-San-Jie (QDSJ) decoction as a therapy for NF2-associated VS. We demonstrated that QDSJ decoction suppresses tumor growth by regulating proliferation, necrotic cell death, apoptosis, and vascular normalization. To our knowledge, this study is the first attempt to evaluate traditional Chinese medicine formulas in the treatment of NF2-associated VS.

## Materials and methods

### Preparation of Qu-Du-San-Jie decoction

QDSJ decoction was prepared from 8 g of *Ginseng Radix Et Rhizoma, 20 g of Gastrodiae Rhizoma, 26 g of Scorpio, 10 g of Scolopendra, 16 g of Chuanxiong Rhizoma, 30 g of Chrysanthemi Indici Flos and 30 g of Hedyotis Diffusae Herba*. Briefly, all the raw materials were co-decocted in water twice for 30 min each time. The amount of solvent used was ten times the total weight of the materials. The decoction was concentrated and a total of approximately 500 ml of decoction was collected. For the animal experiments, the concentrated decoction was used for oral administration. For LC-MS analysis and cell experiments, the concentrated QDSJ decoction was freeze-dried using a Pilot5-8M freeze dryer (Boyikang, Beijing, China) with an extraction yield of 23%. For 125 ml of QDSJ decoction, which was equivalent to the clinical dosage per day, an average amount of 6.69 g of freeze-dried powder was collected.

### Ultra high-performance liquid chromatography-mass spectroscopy analysis

Ten milligrams of freeze-dried QDSJ powder was dissolved in water, sonicated for 30 min, and centrifuged at 1,000 rpm for 5 min. The supernatant was filtered through a 0.22 μm PTFE membrane.

Chromatographic analysis was performed with a Vanquish UHPLC system (Thermo Scientific, Waltham, MA, United States). The samples were separated on a ACQUITY UPLC BEH C18 (3.0 mm × 150 mm i.d. 1.7 µm, Waters, Milford, MA, United States) at a flow rate of 0.4 ml/min and a column temperature of 30°C. The injection volume was 5 μl. The mobile phases consisted of 0.1% aqueous formic acid (A) and acetonitrile solution (B). The gradient elution program was as follows: 90% ∼ 75% B (0 ∼ 10 min), 75% ∼ 60% B (10 ∼ 20 min), 60 ∼ 30% B (20 ∼ 23 min), 30% ∼ 30% B (23 ∼ 26 min), 30% ∼ 10% B (26 ∼ 27 min) and 10% ∼ 10% B (27 ∼ 28 min).

Mass spectrometry was performed using a Q Exactive Plus mass spectrometer (Thermo Scientific, Waltham, MA, United States) equipped with an electrospray ionization source and operated with the Xcalibur (version 2.1) data acquisition software. The experimental conditions for positive ion detection were as follows: mode, HESI ion source; ionization source voltage, 3.5 kV; capillary temperature of 320°C; sheath gas and auxiliary gas, high purity nitrogen (purity >99.99%); sheath gas flow rate, 35 arb; auxiliary gas flow rate, 10 arb. For negative ion detection, the following conditions were applied: mode, HESI ion source; ionization source voltage, 3 kV; capillary temperature of 320°C; sheath gas and the auxiliary gas, high purity nitrogen (purity >99.99%); sheath gas flow rate, 35 arb; and auxiliary gas flow rate, 10 arb. Data of the positive and negative ions were collected by the method of Fourier transform high-resolution full scan (TF, Full scan, Resolution 30,000). Data-dependent acquisition (MS2) was performed by the method of collision-induced dissociation.

The mass spectrometry data were searched with the corresponding information in the OTCML high-resolution mass spectrometry database (Thermo Scientific, Waltham, MA, United States) to identify traditional Chinese medicine components.

### Gene expression profiling

Gene expression profiling analysis was performed on an independent cohort of 37 NF2-associated VSs and seven normal peripheral nerves using the Affymetrix Human Transcriptome Array 2.0 Kit (Affymetrix GeneChip, #902280, Santa Clara, CA, United States), as we described previously (Zhao et al., 2018b; Zhao et al., 2022). Sample library preparation, hybridization, and quality control were performed according to Affymetrix-recommended protocols. The microarray analysis was performed using Affymetrix Expression Console Software (version 1.2.1, Santa Clara, CA, United States). Gene expression profiling data can be acquired from the NCBI GEO database (No. GSE108524). For differentially expressed gene (DEG) analysis, the criteria of fold change ≥ 2 and *p* < 0.05 was used.

### Bioinformatics analyses

The identified compounds in the QDSJ decoction and the Simplified Molecular-Input Line-Entry System (SMILES) strings were retrieved from PubChem (https://pubchem.ncbi.nlm.nih.gov/). The SMILES strings were then submitted to SwissTargetPrediction (http://www.swisstargetprediction.ch) to identify the corresponding targets of QDSJ. Targets with a probability of no less than 0.05 were selected.

To illustrate the relationships between the putative targets of QDSJ and the DEGs of NF2-associated VS, the drug-disease crossover genes were screened. The overlapping genes were submitted to the STRING database (https://string-db.org/) to generate the protein-protein interaction (PPI) network. The PPI network was visualized and analyzed using Cytoscape (version 3.7.2, San Diego, CA, United States). The cytoHubba plug-in was then used to screen the top 15 key genes based on degree scores in the PPI network.

The underlying mechanisms of these overlapping targets and corresponding Gene Ontology (GO) and Kyoto Encyclopedia of Genes and Genomes (KEGG) pathways (https://www.genome.jp/kegg/pathway.htmL) were analyzed using clusterProfiler package (version 4.2) in R software (version 3.6.2).

### Cell line and cell culture

BNI-VS-50 human NF2-associated schwannoma cell line, established from a patient-derived xenograft, was used as we previously described (Zhao et al., 2022). The BNI-VS-50 cells were maintained in Dulbecco’s Modified Eagle Medium (DMEM, Gibco, Grand Island, NY, United States) with 10% fetal bovine serum (FBS, Gibco), and 1% penicillin and streptomycin (Gibco) at 37°C in a humidified incubator containing 5% CO_2_ and routinely passaged every 3 ∼ 4 days.

### Cell viability assay

BNI-VS-50 cells were seeded in 96-well plates at a density of 3,000 cells/well. QDSJ freeze-dried powder was dissolved in PBS and filtered through a 0.22 μm filter. After being treated with QDSJ for 72 h, the cells were observed using a Cytation 5 Cell Imaging multimode reader (BioTek Instruments, Winooski, VT, United States). Cell viability and IC_50_ were analyzed using Gen5 data analysis software (BioTek Instruments GmbH, Bad Friedrichshall, Germany).

### Cell death and apoptosis assays

Calcein-AM/PI staining (CA1630, Solarbio, Beijing, China) was used to assess the cell death of BNI-VS-50 cells after QDSJ treatment. BNI-VS-50 cells were seeded in 24-well plates at a density of 2 × 10^5^ cells/well. After being treated with 250 ng/ml or 350 ng/ml QDSJ or PBS for 48 h, the cells were rinsed in PBS three times and incubated for 20 min in the dark at room temperature in 2 μmol/L Calcein-AM and 5 μmol/L PI. The cells were then gently washed three times in PBS. Excited at 488 nm, the living cells appeared green (per Calcein-AM instructions) and the nuclei of the dead cells displayed red fluorescence (per PI instructions). The stained cells were then imaged under a laser scanning confocal microscope (LSM, Zeiss, Germany).

A pSIVA apoptosis detection kit (Novus Biologicals, Centennial, CO, United States) was used to evaluate cell apoptosis. Treated with QDSJ for 48 h, the BNI-VS-50 cells were incubated with 10 µl/ml of the pSIVA-IANBD probe and 5 µl/ml of PI. Cells were observed under microscope using the green fluorescence filter for pSIVA-IANBD and the red fluorescence filter for PI visualization.

### Western blotting

BNI-VS-50 cells treated with QDSJ or PBS for 48 h were collected and then sonicated in ice-cold RIPA lysis buffer containing a protease and phosphatase inhibitor cocktail (Solarbio, Beijing, China). The cell lysates were centrifuged at 12,000 × g for 15 min at 4°C, and the supernatant was collected. The protein concentrations were determined using the BCA protein quantification assay (Bio-Rad, Cat. # 5000006, Hercules, CA, United States). Twenty micrograms of denatured protein was loaded onto an SDS-PAGE gel, and 5 μl of prestained protein marker was loaded as a molecular weight standard (Solarbio, Beijing, China). The proteins were transferred onto PVDF membranes and incubated with the following primary antibodies: anti-Cyclin D1 (rabbit monoclonal, Cell Signaling Technology, Cat. # 55506S, 1:1,000), and anti-GAPDH (mouse monoclonal, Proteintech, Cat. # 60,004-1-lg, 1:25,000). The membranes were then washed with 0.01% TBS-Triton X and incubated in HRP-conjugated goat anti-rabbit IgG (Cell Signaling Technology, Cat. # 7074S) or goat anti-mouse IgG (Cell Signaling Technology, Cat. # 7076S). The bands were visualized using a chemiluminescence reagent (Thermo Scientific, Cat. # 34580, Waltham, MA, United States). Imaging was performed using Amersham Imager 680 (GE Life Sciences, Pittsburgh, PA, United States). The bands were analyzed with Image Lab Software (Bio-Rad, Hercules, CA, United States).

### Schwannoma xenografts

All the experiments were performed in accordance with the research guidelines set forth by the ethics committee of Beijing Neurosurgical Institute for animal research (N137/10, 201902036). Male BALB/c nude mice (6 weeks old) were purchased from Beijing Vital River Laboratory Animal Technology (Beijing, China). The animals were group-housed and kept on a 12-h light and dark cycle with *ad libitum* access to food and autoclaved water.

BNI-VS-50 cells (1 × 10^6^) were inoculated into the lower backs of BALB/c nude mice using a Hamilton syringe, as we described previously (Zhao et al., 2022). After 14 days, the mice were randomly divided into the QDSJ group and the vehicle group (*n* = 5/group). QDSJ decoction was administered by oral gavage every 12 h for 14 consecutive days. The dose was calculated based on the equivalent dose for humans by multiplying a factor of 0.026 for mice with reference to body surface area. Mice in the vehicle group received autoclaved water instead. Tumor growth was evaluated by monitoring tumor volume every 2 days. The tumor volume was calculated using the following formula: (length × width) × √ (length × width) × (π/6). The animals were sacrificed 24 h after the final administration. The tumor xenografts were harvested for further evaluation.

### Histology and immunostaining

Xenografts were fixed in 4% paraformaldehyde, dehydrated in ethanol, embedded in paraffin, and serially sectioned at 5-μm thickness. For histology, the slides were stained with hematoxylin and eosin.

For immunohistochemistry, antigen retrieval was performed by heating the sections in sodium citrate (pH 6.0) for 18 min. To block endogenous peroxidase activity, the sections were treated with 3% hydrogen peroxide for 15 min. After blocking, the sections were incubated with anti-Cyclin D1 antibody (rabbit monoclonal, Cell Signaling Technology, Cat. # 55506S, 1:500) at 4°C overnight. After washing, the sections were incubated with a biotinylated secondary antibody (Absin, Shanghai, China) for 10 min followed by treatment with HRP, DAB substrate, and hematoxylin counterstaining (Absin, Shanghai, China).

For immunofluorescence staining, after antigen retrieval using sodium citrate buffer, the sections were treated with 0.3% Triton-X-100 in TBS for 30 min and then blocked with TGB superblock containing 10% normal goat serum and 10% BSA. The sections were incubated with primary antibodies at 4°C overnight, including anti-Ki-67 (rabbit polyclonal, Abcam, Cat. # ab15580, 1:1,000), anti-CD31 (rabbit polyclonal, Abcam, Cat. # ab28364, 1:50), and anti-alpha smooth muscle Actin (αSMA, mouse monoclonal, Abcam, Cat. # ab7817, 1:250) antibodies. The secondary antibodies against the appropriate species, including Alexa Fluor 488-conjugated goat anti-rabbit (Jackson ImmunoResearch, Cat. # 111-545-144, 1:300) and Rhodamine Red-X-conjugated goat anti-mouse (Jackson ImmunoResearch, Cat. # 115-295-166, 1:300), were incubated for 2 h at room temperature. The sections were then treated with a True VIEW autofluorescence Quenching Kit (Vector Laboratories, Burlingame, CA, United States) and mounted.

Two sections per xenograft and three random fields per section were used to analyze the results. The sections were 100 μm apart from each other. H&E and IHC staining images were obtained using digital whole slide scanning (APERIO AT2, Leica Biosystems, Buffalo Grove, IL, United States) and reviewed using computer-based image analysis (Image-Pro Plus, Media Cybernetics, MD, United States). Immunofluorescence images were captured using an Olympus BX53 microscope and a DP74 CCD camera (Center Valley, PA, United States). To reduce the variability in signal intensity, images were collected using the same exposure time setting, brightness, and contrast for each fluorophore. The percentages of cells that were positive for Cyclin D1 and Ki-67 expression were measured. The percentage of αSMA-positive vessels, collapsed vessels, and vessel densities were quantified for CD31 staining. The integrated optical densities (IODs) of αSMA were assessed. Images were analyzed using Fiji software (https://imagej.net/Fiji). All the sections were analyzed by an investigator who was blinded to the experimental groups.

### Statistical analysis

Raw data from Fiji and Image Lab analyses were imported into Prism 9 (GraphPad Software, La Jolla, CA, United States) for statistical analyses using ANOVA followed by Tukey’s post-hoc intergroup comparison or using Student’s *t* test. All the data analyzed in this study passed Shapiro-Wilk normality test (*α* = 0.05). IC_50_ values were determined using nonlinear regression analysis of effect-log concentration curves. Graphs were produced in Prism 9, and error bars denote the standard deviation (SDs). Comparisons were interpreted as significant when associated with *p* < 0.05.

## Results

### Chemical compounds and potential targets of Qu-Du-San-Jie decoction

**FIGURE 1:**
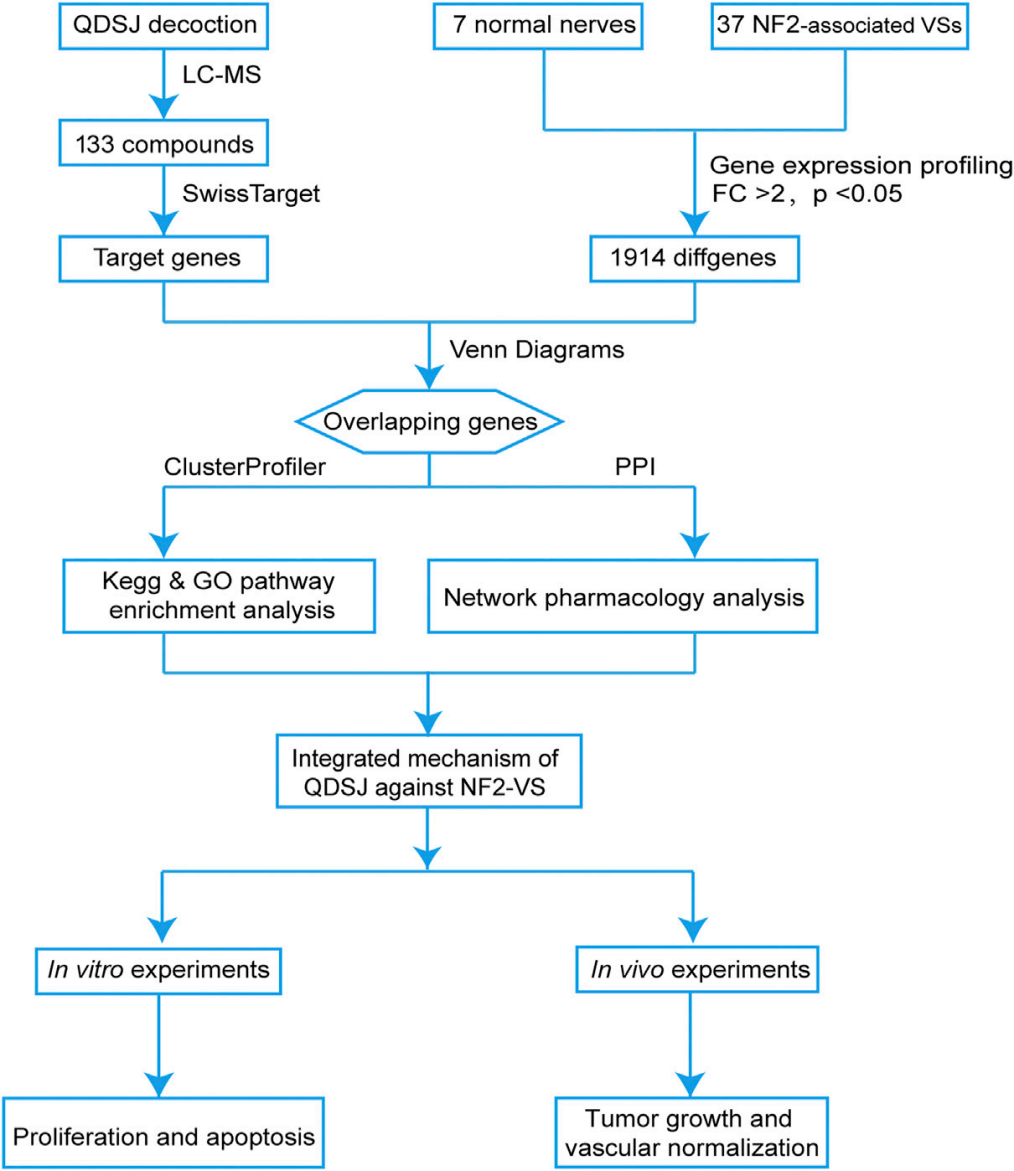
Integrated flowchart for elucidating the mechanism of QDSJ decoction in the treatment of NF2-associated VS. First, a global view of the potential compound-target-pathway network based on UHPLC-MS results for QDSJ decoction and gene expression profiling data from patients was established to predict the potential mechanisms of QDSJ decoction treating NF2-associated VS. Subsequently, *in vitro* and *in vivo* experiments using a patient-derived schwannoma cell line and a xenograft mouse model were conducted to validate the antitumor and antiangiogenic effect of QDSJ decoction treating NF2-associated VS.

**FIGURE 2:**
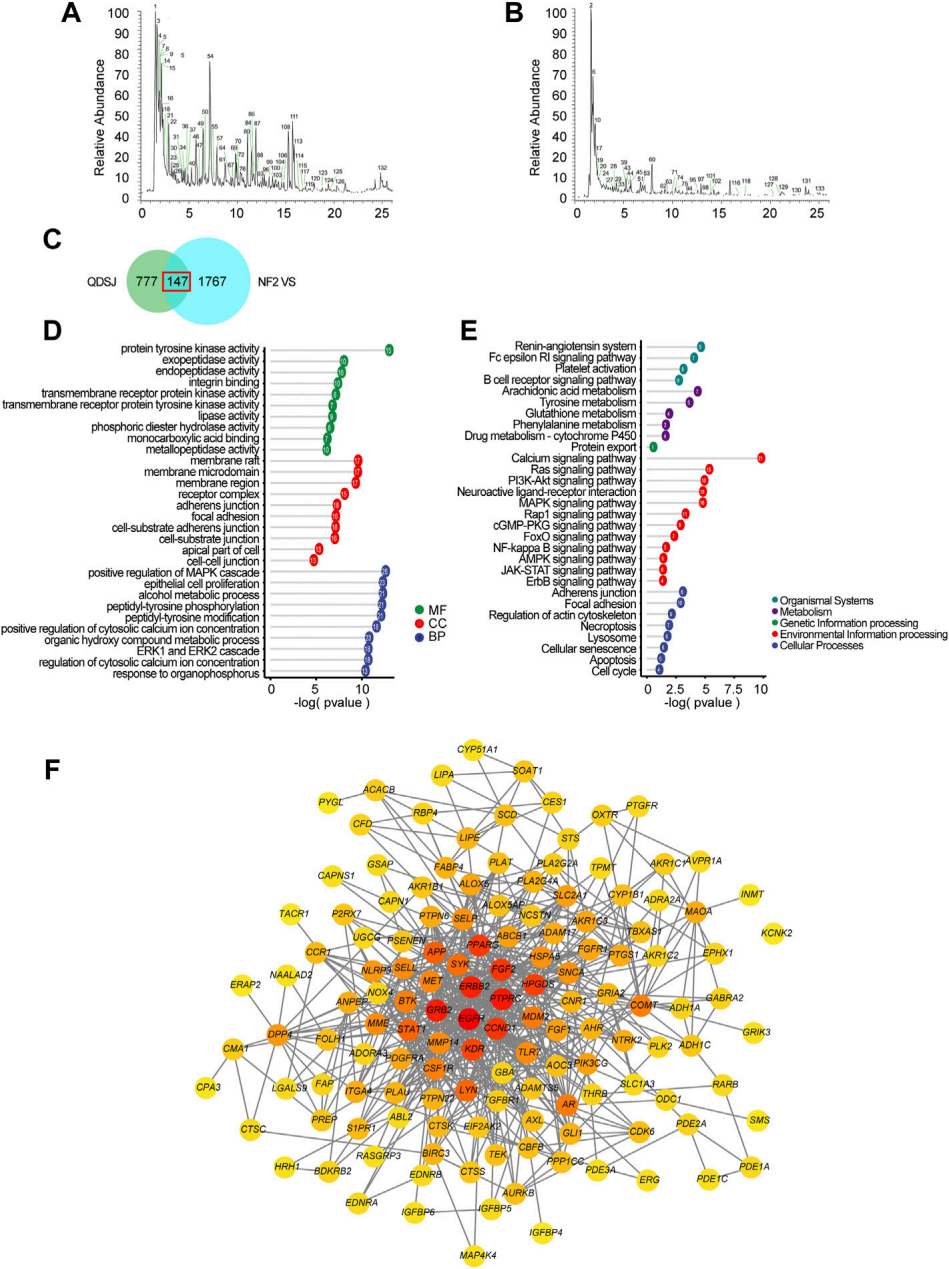
Component-target-pathway analyses of QDSJ decoction in the treatment of NF2-associated VS. **(A)** Positive mode mass spectrum chromatograms of QDSJ decoction. **(B)** Negative mode mass spectrum chromatograms of QDSJ decoction. **(C)** Venn diagram of the drug targets of QDSJ decoction and differentially expressed genes in NF2-associated VS. **(D)** GO terms associated with the overlapping candidate targets of QDSJ in the treatment of NF2-associated VS. The top 10 GO functional categories in molecular function, cellular component, and biological process were selected. **(E)** KEGG pathway enrichment of the overlapping candidate targets of QDSJ in the treatment of NF2-associated VS. **(F)** PPI network of the overlapping genes of QDSJ decoction and NF2-associated VS. The darker color indicates a higher degree. MF: molecular function; CC: cellular component; BP: biological process.

As shown in Figure 1, we performed the component–target–pathway analysis combined with *in vitro* and *in vivo* experiments to reveal the potential mechanism by which the QDSJ decoction affects NF2-associated VS. The chemical constituents of the QDSJ decoction were determined by UHPLC and mass spectrometry (Figures 2A,B). After analyzing the raw data and matching the molecules using the OTCML high-resolution mass spectrometry database, 133 compounds in the QDSJ decoction were identified, including secondary metabolites such as alkaloids, flavonoids, lactones, phenylpropanoids, and carboxylic acids (Supplementary Table S1).

We then collected the potential targets of identified compounds through SwissTargetPrediction. A total of 924 targets were identified after removing duplicates (Supplementary Table S2). To further explore the potential therapeutic targets of QDSJ decoction, we performed GO and pathway enrichment analyses. The top 10 terms of molecular function (MF), cellular component (CC), and biological process (BP) included neurotransmitter receptor activity, component of presynaptic membrane, and regulation of MAPK cascade, which indicated that QDSJ might have therapeutic effects on the central nervous system (Supplementary Figure S1A). Moreover, the results of KEGG enrichment analysis revealed that QDSJ might regulate apoptosis, cell cycle, HIF-1 signaling pathway, VEGF signaling pathway, PI3K-Akt signaling pathway, which are involved in a variety of cancer-related signaling pathways.

### Potential therapeutic targets of Qu-Du-San-Jie decoction on NF2-associated schwannoma

To further elucidate the potential therapeutic effect of QDSJ decoction, the DEGs of NF2-associated VS *versus* normal nerves were identified using the gene expression profiling data we previously collected (GEO No. GSE108524). A total of 1914 DEGs were identified using the criteria of fold change ≥ 2 and *p* < 0.05. Venn diagram analysis showed that QDSJ decoction shared 147 putative targets with NF2-associated VS (Figure 2C). Biological enrichment analysis yielded 1,395 enriched GO terms. The top 10 terms of MF, CC, and BP contained several tumor-related functions, including regulation of MAPK cascade, epithelial cell proliferation, ERK1 and ERK2 cascade, adherens junction, focal adhesion, and growth factor binding (Figure 2D). KEGG pathway analysis showed that potential targets were enriched in 224 signaling pathways, including several key pathways correlated with tumorigenesis and the nervous system, such as necroptosis, apoptosis, cell cycle, adherens junction, neuroactive ligand-receptor interaction, and Ras, PI3K-Akt, MAPK, Rap1, FoxO, NK-κB, and JAK-STAT signaling pathways (Figure 2E). The PPI network is shown in Figure 2F. The median values of DC, EC, LAC, BC, CC, and NC were 8.74, 2.85, 245.76, 0.37, and 4.55, respectively. The top 15 core targets selected through the CytoHubba plug-in were EGFR, CD45, GRB2, HER2, FGF2, Cyclin D1, PPARγ, HPGDS, VEGFR2, STAT1, LYN, SYK, MET, FGF1, and PDGFRA (Supplementary Table S3).

### Qu-Du-San-Jie promoted necrotic cell death and apoptosis of NF2-associated schwannoma cells

**FIGURE 3:**
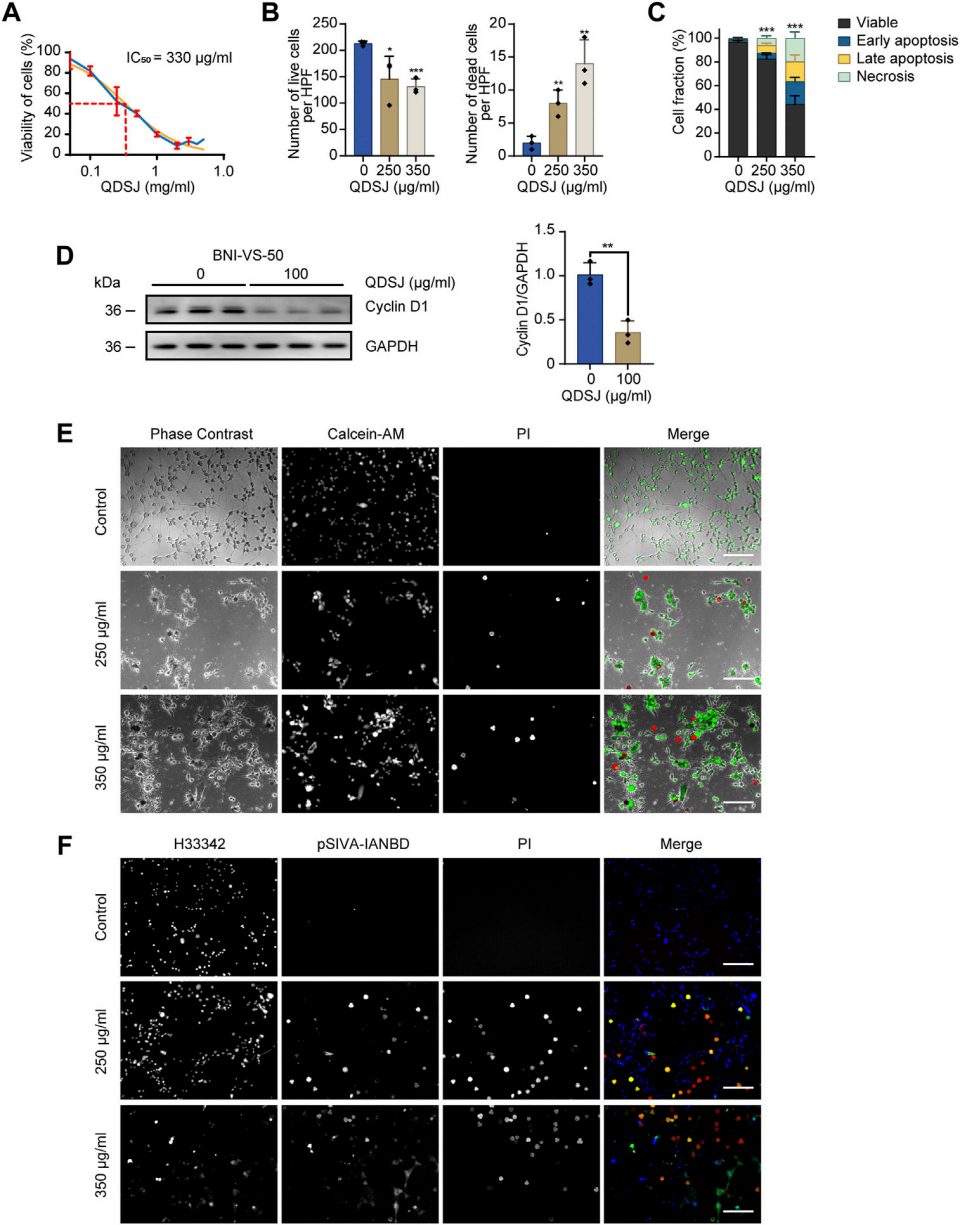
QDSJ decoction induces necrotic cell death and apoptosis in schwannoma cells. **(A)** Dose-response curve of BNI-VS-50 cells treated with freeze-dried QDSJ powder reconstituted in PBS for 72 h. The blue curve represents the experimental data. The orange curve shows the regression curve. IC_50_ =330 μg/ml. **(B)** Numbers of live and dead BNI-VS-50 cells determined by Calcein-AM/PI staining 48 h after QDSJ treatment. **(C)** Percentage of apoptotic and necrotic BNI-VS-50 cells determined by pSIVA-IANBD/PI staining at 48 h after QDSJ treatment. **(D)** Representative blot and quantitative analysis of band optical densities for Cyclin D1 and GAPDH (loading control) from BNI-VS-50 cells collected 48 h after QDSJ treatment. **(E)** Representative images of Calcein-AM/PI staining. Living cells appeared green (Calcein-AM positive) and the nuclei of dead cells emitted red fluorescence (PI positive). **(F)** Representative images of pSIVA-IANBD/PI staining. pSIVA-IANBD negative/PI negative: viable; pSIVA-IANBD positive/PI negative: early apoptosis; pSIVA-IANBD positive/PI positive: late apoptosis; pSIVA-IANBD negative/PI positive: necrosis. Scale bar = 200 μm; *n* = 3/group; means ± SDs; ^*^ *p* < 0.05, ^**^ *p* < 0.01, and ^***^ *p* < 0.001 compared to the control group by ANOVA followed by Tukey’s post hoc-test **(B)**, 3-way ANOVA **(C)**, and Student’s *t* test **(D)**. HPF: high-power field.

Human NF2-associated schwannoma cell line BNI-VS-50 established from patient-derived xenograft was used to evaluate the antitumor potential of QDSJ decoction. The QDSJ decoction was freeze-dried and 5.352 g of QDSJ powder was obtained from every 100 ml decoction. Freeze-dried QDSJ powder was dissolved in PBS, sonicated, centrifuged, and filtered through a 0.22 μm PTFE membrane. We found that QDSJ significantly reduced the viability of BNI-VS-50 cells after 72 h of treatment, with an IC_50_ of 330 μg/ml (Figure 3A).

To determine whether QDSJ induces cell death, we performed Calcein-AM/PI staining of BNI-VS-50 cells 48 h after QDSJ treatment. As expected, the numbers of Calcein-AM-positive live cells were decreased by 31.61% and 38.34%, and the numbers of PI-positive dead cells were 4.0- and 7.0-fold higher than those in the control group when the cells were treated with 250 μg/ml and 350 μg/ml QDSJ, respectively (*p* < 0.05, Figures 3B,E). To further confirm whether the observed cell death was caused by necrosis or apoptosis, pSIVA-IANBD/PI staining was performed. The numbers of early apoptotic cells, late apoptotic cells, and necrotic cells were 2.18-, 10.49-, and 9.41-fold higher, respectively, compared to the control group 48 h after 250 μg/ml QDSJ treatment (*p* < 0.01); and the numbers of early apoptotic cells, late apoptotic cells, and necrotic cells were 8.84-, 27.01-, and 30.06-fold higher, respectively, compared to control group 48 h after 350 μg/ml QDSJ treatment (*p* < 0.001, Figures 3C,F). The expression level of Cyclin D1 was significantly decreased 48 h after 100 μg/ml QDSJ treatment (*p* < 0.01, Figure 3D).

### Qu-Du-San-Jie decoction suppressed tumor growth of NF2-associated vestibular schwannoma *in vivo*

**FIGURE 4:**
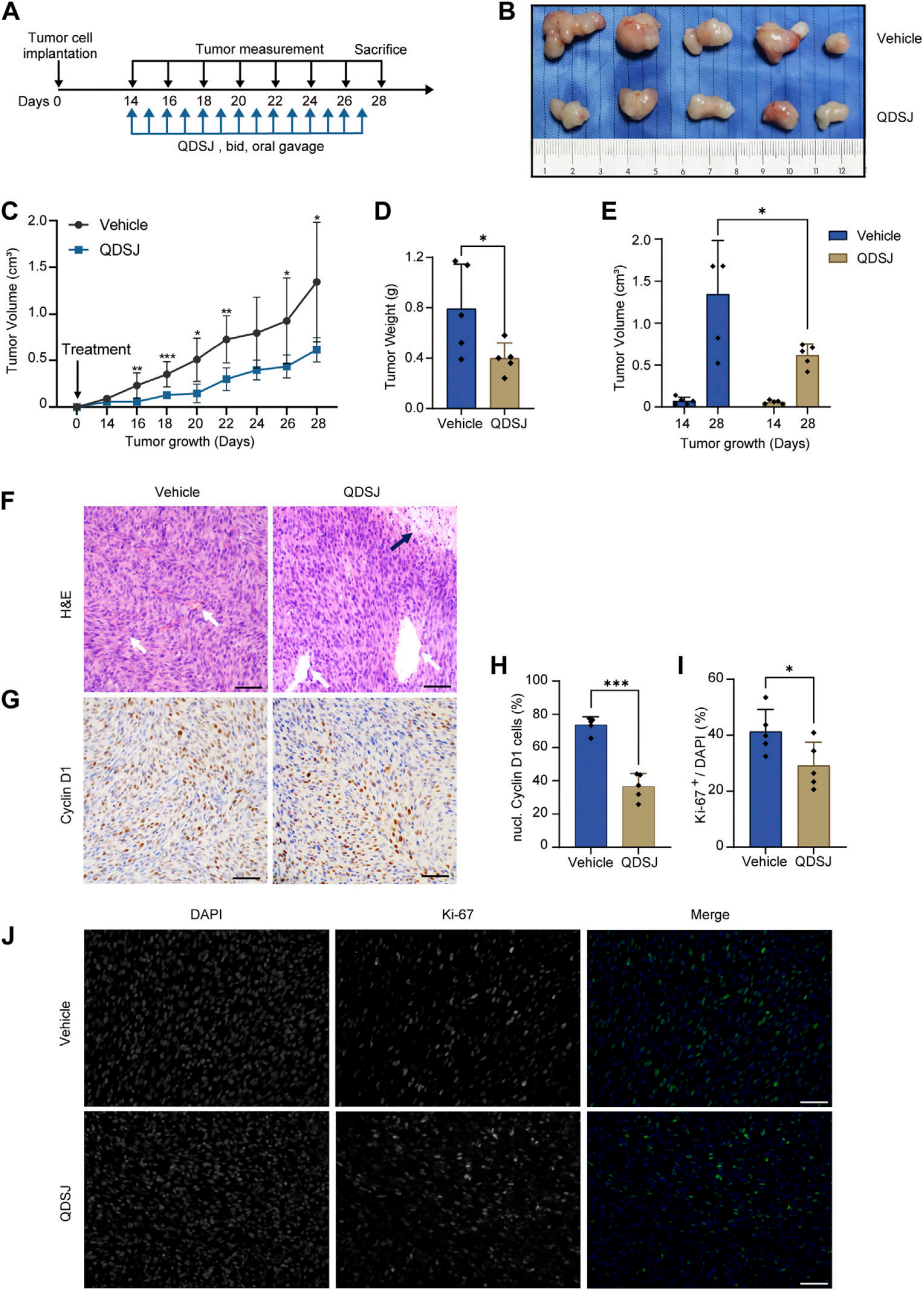
Treatment with QDSJ decoction suppresses schwannoma growth *in vivo.* **(A)** Outline of the experimental paradigm of BNI-VS-50 cell implantation and QDSJ treatment. QDSJ decoction was administered by oral gavage twice a day for 2 weeks. **(B)** Xenografts from the vehicle group and the QDSJ group. **(C)** Tumor growth curves of xenografts after BNI-VS-50 cell implantation and QDSJ treatment. Tumor sizes were measured every 2 days. A marked decrease in tumor size was observed 2 days after QDSJ treatment. **(D)** Tumor weight of the vehicle- and the QDSJ-treated groups 24 h after final administration. **(E)** Tumor volume of the vehicle- and QDSJ-treated groups 14 days (before QDSJ treatment) and 28 days (after QDSJ treatment) after BNI-VS-50 implantation. **(F)** Representative images of H&E staining of the xenografts. Enlarged vascular vessels (white arrow) and a large area of cell necrosis were observed in QDSJ-treated tumors (black arrow). **(G)** Representative images of Cyclin D1 immunohistochemistry staining of the xenografts. **(H)** The fraction of Cyclin D1-positive nuclei in the vehicle- and QDSJ-treated groups. **(I)** The fraction of Ki-67-positive cells in the vehicle- and QDSJ-treated groups. **(J)** Representative images of Ki-67 immunofluorescence staining of xenografts in QDSJ- and vehicle-treated tumors. Scale bar = 100 μm; *n* = 5/group; means ± SDs; ^*^ *p* < 0.05, ^**^ *p* < 0.01, and ^***^ *p* < 0.001 by Student’s *t* test.

As QDSJ improved the necrosis and apoptosis and inhibited the proliferation of BNI-VS-50 cells, we evaluated the antitumor effect of QDSJ decoction on NF2-associated VS *in vivo*. An NF2-associated VS xenograft mouse model was established using BNI-VS-50 cells. QDSJ decoction was administered by oral gavage every 12 h for 14 consecutive days with the equivalent dose as clinical use for humans, which multiplied a factor of 0.026 with reference to body surface area (Figure 4A). We observed that oral treatment with QDSJ decoction significantly inhibited tumor growth (Figure 4B). The tumor volume was reduced by 54.11% (1.34 ± 0.64 cm^3^ in vehicle-treated mice and 0.62 ± 0.13 cm^3^ in QDSJ-treated mice), and the tumor weight was reduced by 49.74% (0.79 ± 0.35 g in vehicle-treated mice and 0.40 ± 0.12 g in QDSJ-treated mice) after 14 days of QDSJ treatment compared to vehicle treatment (*p* < 0.05, Figures 4C–E). No systemic toxicity (loss of appetite or bodyweight loss) was observed in any mouse (Supplementary Figure S2). H&E staining showed cell necrosis areas and enlarged tumor blood vessels in QDSJ-treated tumors (Figure 4F). IHC analysis showed that the percentage of Cyclin D1-positive cells decreased significantly in QDSJ-treated tumors (*p* < 0.001, Figures 4G,H). Immunofluorescence staining showed lower Ki-67 indices in QDSJ-treated tumors compared to vehicle-treated tumors (*p* < 0.05, Figures 4I,J).

### Qu-Du-San-Jie decoction normalized tumor vasculature of NF2-associated vestibular schwannoma *in vivo*

**FIGURE 5:**
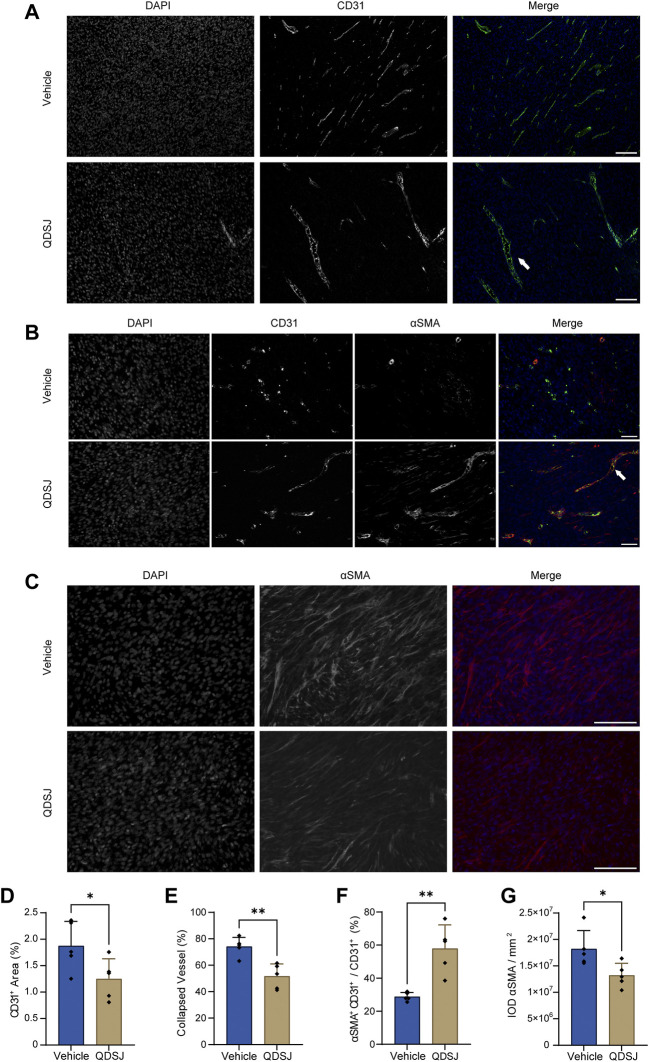
Treatment with QDSJ decoction reduces the number and density of vessels and normalizes vessel structure in schwannoma. **(A–C)** Representative immunofluorescence staining of CD31 (an endothelial cell marker, green) and αSMA (a pericyte and fibrosis marker, red) in QDSJ- and vehicle-treated tumors. Enlarged vascular vessels (white arrow in A) and pericyte-covered vessels (white arrow in B) were observed in the QDSJ-treated tumors. The fraction of CD31-positive vessel area **(D)**, collapsed vessels **(E)**, pericyte-covered vessels (% αSMA+CD31+/CD31+) **(F)** and IOD of αSMA fluorescence **(G)** were quantified by using ImageJ software. Scale bar = 100 μm **(A–C)**; *n* = 5/group; means ± SDs; ^*^ *p* < 0.05, and ^**^ *p* < 0.01 by Student’s *t* test.

Impressed with the suppressed tumor growth and cell proliferation after QDSJ treatment, and the tumor angiogenesis-related proteins, including EGFR, FGF1, FGF2, VEGRR2, and PDGFRA revealed in the top 15 key targets in the PPI network of QDSJ decoction treating NF2-associated VS, we hypothesized that QDSJ decoction would attenuate tumor angiogenesis of the NF2-associated VS. As expected, decreased vessel density after QDSJ treatment was confirmed by evaluating the areas covered by vessels (*p* < 0.05, Figures 5A,D). Next, to determine whether structural normalization was accompanied by the anti-angiogenesis effect of QDSJ since pericyte detachment of tumor vessels leads to abnormal vessel perfusion (Gao et al., 2015; Viallard and Larrivee, 2017), we performed CD31/αSMA staining to assess the pericyte coverage. We found that the percentage of αSMA^+^/CD31^+^ cells, which indicated the pericyte-covered vessels, was significantly increased after QDSJ treatment compared to the vehicle-treated group (28.77% ± 2.58% in vehicle-treated mice and 57.90% ± 14.36% in QDSJ-treated mice, *p* < 0.01, Figures 5B,F).

Vessel collapse is another characteristic of tumor vasculature, which might be exacerbated by tumor fibrosis, leading to interruption of blood flow and hypoperfused hypoxic areas (Viallard and Larrivee, 2017; Wu L. et al., 2021). CD31 staining showed that the ratios of collapsed vessels were significantly decreased after QDSJ treatment (74.09% ± 6.96% in vehicle-treated mice and 51.60% ± 9.35% in QDSJ-treated mice, *p* < 0.01, Figures 5A,E). The integrated optical densities of αSMA (as fibrosis marker) were significantly decreased in QDSJ-treated mice compared to vehicle-treated mice (*p* < 0.05, Figures 5C,G).

## Discussion

The clinical treatment of NF2-associated schwannoma is still a challenge due to the high risk of morbidity (Zhao et al., 2018a). The current therapeutic strategies offer limited benefits. The only effective drug, bevacizumab, has been demonstrated in some but not all NF2 patients, and some problems, such as side effects, still exist (Plotkin et al., 2009; Plotkin et al., 2019). Recently, clinical trials of TCM have previously been conducted to assess the safety, tolerability, and potential clinical activity in patients with brain tumors (Efferth et al., 2017; Wang et al., 2020; Wu J. et al., 2021). QDSJ decoction, a TCM formula, has been used as a treatment for multiple cancers including glioblastoma, lung adenocarcinoma, and hepatocellular carcinoma over the past decades, but to our knowledge, no double-blind placebo-controlled trials or preclinical experiments have been carried out for these indications. To test the antitumor efficacy of QDSJ decoction, we employed our patient-derived NF2-associated schwannoma cell line and xenograft models. We found *in vitro* and *in vivo* antitumor effects of QDSJ decoction on NF2-associated VS. Our findings indicate that QDSJ decoction may have the potential capacity to slow the growth of VSs in NF2 patients.

In recent years there has been intense interest in developing chemotherapeutic strategies for patients with NF2 tumors (Widemann et al., 2013). TCM has a unique and integrative theoretical system for strengthening body health, expelling pathogens, and suppressing tumors (Wang et al., 2020; Wang et al., 2021). It was shown that TCM could have advantages in improving the quality of life of patients with cancers in some cases (Qi et al., 2015). Our study is to the best of our knowledge the first to evaluate the antitumor effect of Chinese medicine formula in the treatment of schwannoma. QDSJ is a Chinese medicine formula that exerts antitumor effects on schwannoma. In this study, we further evaluated the efficacy of QDSJ decoction in treating NF2-associated VS and explored the underlying mechanism.

TCM has been used in treating tumors relying on its multi-target characteristics targeting several crucial proteins in cancer-related signaling networks (Wang et al., 2020). Tumor suppressor protein merlin encoded by the NF2 gene is responsible for membrane stabilization and the regulation of several cellular growth pathways (Rouleau et al., 1993). Loss of merlin/NF2 leads to the abnormal activation of multiple pathways, including PI3K/Akt, MET, MAPK, and Hippo-YAP pathways (Okada et al., 2009; Wu L. et al., 2021). Thus, the inhibition of a single target or signaling pathway may have limited therapeutic effects on NF2-associated VS (Sagers et al., 2020). Thus, novel multitargeted therapies are urgently needed. The bioinformatics analyses using data from LC-MS and gene expression profiling indicate that QDSJ might have an antitumor effect on NF2-associated VS by regulating necroptosis, apoptosis, cell cycle, adherens junction, and Ras, PI3K-Akt, MAPK, MET, FoxO, NK-κB, and JAK-STAT signaling pathways. Moreover, we found that QDSJ decoction suppresses the growth of the NF2-associated VS cell line and mouse xenografts by inhibiting cell proliferation, improving apoptosis, and regulating cell cycle.

Tumor blood vessels are a key target for inhibiting tumor progression. Poor vessel functionality caused by the improper development of tumor blood vessels leads to poor hypoxia, decreased immune cell infiltration and activity, and increased risks of metastatic dissemination (Viallard and Larrivee, 2017). Antiangiogenic therapies can normalize the structural and functional flaws, restore the hypoxic microenvironment, and enhance the benefits of antitumor drugs which rely on adequate tumor blood flow for drug delivery (Jain, 2005; Goel et al., 2011; Jain, 2014). Several preclinical and clinical trials have demonstrated that antiangiogenic therapies can inhibit NF2-associated VS tumor growth and postpone the related hearing loss (Wong et al., 2010; Gao et al., 2015; Killeen et al., 2019; Renzi et al., 2020). However, responses to these drugs, such as Bevacizumab, are not durable, suggesting that either longer maintenance therapy or new strategies are required (Nigro et al., 2020; Renzi et al., 2020). Our bioinformatic analysis showed that tumor angiogenesis-related proteins, including EGFR, FGF1, FGF2, VEGRR2, and PDGFRA, were in the top 15 key targets in the PPI network of QDSJ decoction treating NF2-associated VS. Our immunofluorescence staining results confirmed that the tumor vessel densities and the fractions of collapsed vessels were decreased, while the fractions of pericyte-covered vessels were increased after QDSJ treatment, suggesting that QDSJ could reduce angiogenesis and improve tumor vascular normalization in NF2-associated VS xenografts.

This study revealed that QDSJ decoction exerts a multitarget antitumor effect on NF2-associated VS by increasing cell apoptosis, reducing cell proliferation, and normalizing tumor blood vasculature. The bioinformatics analyses suggested the involvement of several signaling pathways that are closely related to the nervous system and tumorigenesis. As our study is the first to validate the effect and mechanism of a Chinese medicine formula in treating NF2-associated schwannoma, further pharmacological and pharmacokinetic studies will be needed to understand the mechanisms and biological efficacy of QDSJ to optimize this therapeutic strategy.

In summary, we show that QDSJ decoction suppresses tumor growth and improves vascular normalization of NF2-associated VS. Furthermore, the drug-target-pathway network elucidated by bioinformatics analyses illustrated the potential mechanisms by which QDSJ decoction affects NF2-associated VS. These results suggest that QDSJ could be a new strategy for the treatment of schwannoma.

## Data Availability

The datasets presented in this study can be found in online repositories. The names of the repository/repositories and accession number(s) can be found in the article/Supplementary Material.

## References

[B1] Ammoun S. Schmid M. C. Triner J. Manley P. Hanemann C. O. (2011). Nilotinib alone or in combination with selumetinib is a drug candidate for neurofibromatosis type 2. Neuro. Oncol. 13 (7), 759–766. 10.1093/neuonc/nor056 21727212 PMC3129276

[B2] Asthagiri A. R. Parry D. M. Butman J. A. Kim H. J. Tsilou E. T. Zhuang Z. (2009). Neurofibromatosis type 2. Lancet 373 (9679), 1974–1986. 10.1016/S0140-6736(09)60259-2 19476995 PMC4748851

[B3] Brackmann D. E. Fayad J. N. Slattery W. H. 3rd Friedman R. A. Day J. D. Hitselberger W. E. (2001). Early proactive management of vestibular schwannomas in neurofibromatosis type 2. Neurosurgery 49 (2), 274–280. 10.1097/00006123-200108000-00007 11504103

[B4] Dirks M. S. Butman J. A. Kim H. J. Wu T. Morgan K. Tran A. P. (2012). Long-term natural history of neurofibromatosis Type 2-associated intracranial tumors. J. Neurosurg. 117 (1), 109–117. 10.3171/2012.3.JNS111649 22503123 PMC4749021

[B5] Efferth T. Schottler U. Krishna S. Schmiedek P. Wenz F. Giordano F. A. (2017). Hepatotoxicity by combination treatment of temozolomide, artesunate and Chinese herbs in a glioblastoma multiforme patient: case report review of the literature. Arch. Toxicol. 91 (4), 1833–1846. 10.1007/s00204-016-1810-z 27519711

[B6] Evans D. G. Huson S. M. Donnai D. Neary W. Blair V. Teare D. (1992). A genetic study of type 2 neurofibromatosis in the United Kingdom. I. Prevalence, mutation rate, fitness, and confirmation of maternal transmission effect on severity. J. Med. Genet. 29 (12), 841–846. 10.1136/jmg.29.12.841 1479598 PMC1016198

[B7] Forde C. King A. T. Rutherford S. A. Hammerbeck-Ward C. Lloyd S. K. Freeman S. R. (2021). Disease course of neurofibromatosis type 2: a 30-year follow-up study of 353 patients seen at a single institution. Neuro. Oncol. 23 (7), 1113–1124. 10.1093/neuonc/noaa284 33336705 PMC8248850

[B8] Fujii M. Ichikawa M. Iwatate K. Bakhit M. Yamada M. Kuromi Y. (2020). Bevacizumab therapy of neurofibromatosis type 2 associated vestibular schwannoma in Japanese patients. Neurol. Med. Chir. 60 (2), 75–82. 10.2176/nmc.oa.2019-0194 PMC7040431 31902875

[B9] Gao X. Zhao Y. Stemmer-Rachamimov A. O. Liu H. Huang P. Chin S. (2015). Anti-VEGF treatment improves neurological function and augments radiation response in NF2 schwannoma model. Proc. Natl. Acad. Sci. U. S. A. 112 (47), 14676–14681. 10.1073/pnas.1512570112 26554010 PMC4664377

[B10] Goel S. Duda D. G. Xu L. Munn L. L. Boucher Y. Fukumura D. (2011). Normalization of the vasculature for treatment of cancer and other diseases. Physiol. Rev. 91 (3), 1071–1121. 10.1152/physrev.00038.2010 21742796 PMC3258432

[B11] Goldbrunner R. Weller M. Regis J. Lund-Johansen M. Stavrinou P. Reuss D. (2020). EANO guideline on the diagnosis and treatment of vestibular schwannoma. Neuro. Oncol. 22 (1), 31–45. 10.1093/neuonc/noz153 31504802 PMC6954440

[B12] Goutagny S. Raymond E. Esposito-Farese M. Trunet S. Mawrin C. Bernardeschi D. (2015). Phase II study of mTORC1 inhibition by everolimus in neurofibromatosis type 2 patients with growing vestibular schwannomas. J. Neurooncol. 122 (2), 313–320. 10.1007/s11060-014-1710-0 25567352

[B13] Jain R. K. (2014). Antiangiogenesis strategies revisited: from starving tumors to alleviating hypoxia. Cancer Cell 26 (5), 605–622. 10.1016/j.ccell.2014.10.006 25517747 PMC4269830

[B14] Jain R. K. (2005). Normalization of tumor vasculature: an emerging concept in antiangiogenic therapy. Science 307 (5706), 58–62. 10.1126/science.1104819 15637262

[B15] Karajannis M. A. Legault G. Hagiwara M. Ballas M. S. Brown K. Nusbaum A. O. (2012). Phase II trial of lapatinib in adult and pediatric patients with neurofibromatosis type 2 and progressive vestibular schwannomas. Neuro. Oncol. 14 (9), 1163–1170. 10.1093/neuonc/nos146 22844108 PMC3424212

[B16] Killeen D. E. Klesse L. Tolisano A. M. Hunter J. B. Kutz J. W. Jr. (2019). Long-Term effects of bevacizumab on vestibular schwannoma volume in neurofibromatosis type 2 patients. J. Neurol. Surg. B Skull Base 80 (5), 540–546. 10.1055/s-0038-1676628 31534897 PMC6748845

[B17] Li S. W. Zhang J. Tang H. L. Li P. Wang B. Zhao F. (2021). Establishment of nomograms for the prediction of useful hearing loss in patients with neurofibromatosis type 2. J. Neurooncol. 155 (3), 373–381. 10.1007/s11060-021-03889-2 34751884

[B18] Maducdoc M. M. Ghavami Y. Linskey M. E. Djalilian H. R. (2015). Evaluation of reported malignant transformation of vestibular schwannoma: De novo and after stereotactic radiosurgery or surgery. Otol. Neurotol. 36 (8), 1301–1308. 10.1097/MAO.0000000000000801 26134937

[B19] Mathieu D. Kondziolka D. Flickinger J. C. Niranjan A. Williamson R. Martin J. J. (2007). Stereotactic radiosurgery for vestibular schwannomas in patients with neurofibromatosis type 2: an analysis of tumor control, complications, and hearing preservation rates. Neurosurgery 60 (3), 460–468. 10.1227/01.NEU.0000255340.26027.53 17327790

[B20] Mautner V. F. Nguyen R. Kutta H. Fuensterer C. Bokemeyer C. Hagel C. (2010). Bevacizumab induces regression of vestibular schwannomas in patients with neurofibromatosis type 2. Neuro. Oncol. 12 (1), 14–18. 10.1093/neuonc/nop010 20150363 PMC2940556

[B21] Morris K. A. Golding J. F. Blesing C. Evans D. G. Ferner R. E. Foweraker K. (2017). Toxicity profile of bevacizumab in the UK Neurofibromatosis type 2 cohort. J. Neurooncol. 131 (1), 117–124. 10.1007/s11060-016-2276-9 27796735

[B22] Nigro O. Coppola A. Tartaro T. Tuzi A. Vallini I. Pinotti G. (2020). Long-term therapy with bevacizumab in a young patient affected by NF2. Stop or continue treatment? An update of a case report and review of the literature. Anticancer. Drugs 31 (7), 754–757. 10.1097/CAD.0000000000000953 32697470

[B23] Okada M. Wang Y. Jang S. W. Tang X. Neri L. M. Ye K. (2009). Akt phosphorylation of merlin enhances its binding to phosphatidylinositols and inhibits the tumor-suppressive activities of merlin. Cancer Res. 69 (9), 4043–4051. 10.1158/0008-5472.CAN-08-3931 19351837 PMC2810124

[B24] Phi J. H. Kim D. G. Chung H. T. Lee J. Paek S. H. Jung H. W. (2009). Radiosurgical treatment of vestibular schwannomas in patients with neurofibromatosis type 2: tumor control and hearing preservation. Cancer 115 (2), 390–398. 10.1002/cncr.24036 19109818

[B25] Plotkin S. R. Duda D. G. Muzikansky A. Allen J. Blakeley J. Rosser T. (2019). Multicenter, prospective, phase II and biomarker study of high-dose bevacizumab as induction therapy in patients with neurofibromatosis type 2 and progressive vestibular schwannoma. J. Clin. Oncol. 37 (35), 3446–3454. 10.1200/JCO.19.01367 31626572 PMC7098833

[B26] Plotkin S. R. Halpin C. McKenna M. J. Loeffler J. S. Batchelor T. T. Barker F. G. 2nd (2010). Erlotinib for progressive vestibular schwannoma in neurofibromatosis 2 patients. Otol. Neurotol. 31 (7), 1135–1143. 10.1097/MAO.0b013e3181eb328a 20736812 PMC4030413

[B27] Plotkin S. R. Stemmer-Rachamimov A. O. Barker F. G. 2nd Halpin C. Padera T. P. Tyrrell A. (2009). Hearing improvement after bevacizumab in patients with neurofibromatosis type 2. N. Engl. J. Med. 361 (4), 358–367. 10.1056/NEJMoa0902579 19587327 PMC4816642

[B28] Qi F. Zhao L. Zhou A. Zhang B. Li A. Wang Z. (2015). The advantages of using traditional Chinese medicine as an adjunctive therapy in the whole course of cancer treatment instead of only terminal stage of cancer. Biosci. Trends 9 (1), 16–34. 10.5582/bst.2015.01019 25787906

[B29] Renzi S. Michaeli O. Salvador H. Alderete D. Ponce N. F. Zapotocky M. (2020). Bevacizumab for NF2-associated vestibular schwannomas of childhood and adolescence. Pediatr. Blood Cancer 67 (5), e28228. 10.1002/pbc.28228 32124552

[B30] Rouleau G. A. Merel P. Lutchman M. Sanson M. Zucman J. Marineau C. (1993). Alteration in a new gene encoding a putative membrane-organizing protein causes neuro-fibromatosis type 2. Nature 363 (6429), 515–521. 10.1038/363515a0 8379998

[B31] Sagers J. E. Beauchamp R. L. Zhang Y. Vasilijic S. Wu L. DeSouza P. (2020). Combination therapy with mTOR kinase inhibitor and dasatinib as a novel therapeutic strategy for vestibular schwannoma. Sci. Rep. 10 (1), 4211. 10.1038/s41598-020-60156-6 32144278 PMC7060236

[B32] Samii M. Matthies C. Tatagiba M. (1997). Management of vestibular schwannomas (acoustic neuromas): auditory and facial nerve function after resection of 120 vestibular schwannomas in patients with neurofibromatosis 2. Neurosurgery 40 (4), 696–705. 10.1097/00006123-199704000-00007 9092842

[B33] Shinya Y. Hasegawa H. Shin M. Sugiyama T. Kawashima M. Takahashi W. (2019). Long-Term outcomes of stereotactic radiosurgery for vestibular schwannoma associated with neurofibromatosis type 2 in comparison to sporadic schwannoma. Cancers (Basel) 11 (10), E1498. 10.3390/cancers11101498 31591325 PMC6827030

[B34] Slusarz K. M. Merker V. L. Muzikansky A. Francis S. A. Plotkin S. R. (2014). Long-term toxicity of bevacizumab therapy in neurofibromatosis 2 patients. Cancer Chemother. Pharmacol. 73 (6), 1197–1204. 10.1007/s00280-014-2456-2 24710627

[B35] Tamura R. Tanaka T. Miyake K. Yoshida K. Sasaki H. (2017). Bevacizumab for malignant gliomas: current indications, mechanisms of action and resistance, and markers of response. Brain Tumor Pathol. 34 (2), 62–77. 10.1007/s10014-017-0284-x 28386777

[B36] Viallard C. Larrivee B. (2017). Tumor angiogenesis and vascular normalization: alternative therapeutic targets. Angiogenesis 20 (4), 409–426. 10.1007/s10456-017-9562-9 28660302

[B37] Wang J. Qi F. Wang Z. Zhang Z. Pan N. Huai L. (2020). A review of traditional Chinese medicine for treatment of glioblastoma. Biosci. Trends 13 (6), 476–487. 10.5582/bst.2019.01323 31866614

[B38] Wang K. Chen Q. Shao Y. Yin S. Liu C. Liu Y. (2021). Anticancer activities of TCM and their active components against tumor metastasis. Biomed. Pharmacother. 133, 111044. 10.1016/j.biopha.2020.111044 33378952

[B39] Widemann B. C. Blakeley J. O. Dombi E. Fisher M. J. Hanemann C. O. Walsh K. S. (2013). Conclusions and future directions for the REiNS international collaboration. Neurology 81 (21), S41–S44. 10.1212/01.wnl.0000435748.79908.c5 24249805 PMC3908339

[B40] Wong H. K. Lahdenranta J. Kamoun W. S. Chan A. W. McClatchey A. I. Plotkin S. R. (2010). Anti-vascular endothelial growth factor therapies as a novel therapeutic approach to treating neurofibromatosis-related tumors. Cancer Res. 70 (9), 3483–3493. 10.1158/0008-5472.CAN-09-3107 20406973 PMC4785015

[B41] Wu J. Xie S. Li H. Zhang Y. Yue J. Yan C. (2021). Antitumor effect of IL-12 gene-modified bone marrow mesenchymal stem cells combined with Fuzheng Yiliu decoction in an *in vivo* glioma nude mouse model. J. Transl. Med. 19 (1), 143. 10.1186/s12967-021-02809-2 33827606 PMC8028710

[B42] Wu L. Vasilijic S. Sun Y. Chen J. Landegger L. D. Zhang Y. (2021). Losartan prevents tumor-induced hearing loss and augments radiation efficacy in NF2 schwannoma rodent models. Sci. Transl. Med. 13 (602), eabd4816. 10.1126/scitranslmed.abd4816 34261799 PMC8409338

[B43] Zhao F. Chen Y. Li S. W. Zhang J. Zhang S. Zhao X. B. (2022). Novel patient-derived xenograft and cell line models for therapeutic screening in NF2-associated schwannoma. J. Pathol. 257, 620–634. 10.1002/path.5908 35394061

[B44] Zhao F. Wang B. Yang Z. Zhou Q. Li P. Wang X. (2018a). Surgical treatment of large vestibular schwannomas in patients with neurofibromatosis type 2: outcomes on facial nerve function and hearing preservation. J. Neurooncol. 138 (2), 417–424. 10.1007/s11060-018-2812-x 29492767

[B45] Zhao Y. Liu P. Zhang N. Chen J. Landegger L. D. Wu L. (2018b). Targeting the cMET pathway augments radiation response without adverse effect on hearing in NF2 schwannoma models. Proc. Natl. Acad. Sci. U. S. A. 115 (9), E2077–e2084. 10.1073/pnas.1719966115 29440379 PMC5834719

