# Supplementary material for: Qu-Du-San-Jie decoction induces growth inhibition and vascular normalization in NF2-associated vestibular schwannoma
Source: Front Pharmacol. 2022 Aug 19;13:941854. doi: 10.3389/fphar.2022.941854 (PMC9437245; doi:10.3389/fphar.2022.941854)
Supplement: Supplementary file 2 [file Table1.docx]

Table S1. Compounds identified from QDSJ Decoction by UHPLC-MS analysis

| **No.** | **+/-** | **RT (min)** | **Ion (m/z)** | **Molecular formula** | **Identity** |
| --- | --- | --- | --- | --- | --- |
| 1 | + | 1.64 | 162.11 | C_7_H_15_NO_3_ | L(-)-Carnitine |
| 2 | - | 1.66 | 191.06 | C_7_H_12_O_6_ | Quinic acid |
| 3 | + | 1.67 | 148.06 | C_5_H_9_NO_4_ | L-Glutamic acid |
| 4 | + | 1.70 | 152.06 | C_5_H_5_N_5_O | Guanine |
| 5 | + | 1.70 | 138.06 | C_7_H_8_NO_2_Cl | Trigonelline HCl |
| 6 | - | 1.87 | 191.02 | C_6_H_8_O_7_ | Citric acid |
| 7 | + | 1.89 | 124.04 | C_6_H_5_NO_2_ | Nicotinic acid |
| 8 | + | 1.92 | 123.06 | C_6_H_6_N_2_O | Nicotinamide |
| 9 | + | 1.95 | 268.1 | C_10_H_13_N_5_O_4_ | Adenosine |
| 10 | - | 2.03 | 243.06 | C_9_H_12_N_2_O_6_ | Uridine |
| 11 | + | 2.04 | 408.15 | C_16_H_22_O_11_ | Deacetylasperulosidic acid |
| 12 | + | 2.07 | 284.1 | C_10_H_13_N_5_O_5_ | Guanosine |
| 13 | + | 2.10 | 269.09 | C_10_H_12_N_4_O_5_ | Inosine |
| 14 | + | 2.14 | 121.07 | C_8_H_8_O | Acetophenone |
| 15 | + | 2.14 | 182.08 | C_9_H_11_NO_3_ | L-Tyrosine |
| 16 | + | 2.17 | 127.04 | C_6_H_6_O_3_ | 5-Hydroxymethylfurfural |
| 17 | - | 2.21 | 331.1 | C_13_H_18_O_7_ | D(-)-Salicin |
| 18 | + | 2.38 | 221.09 | C_11_H_12_N_2_O_3_ | 5-Hydroxytryptophan |
| 19 | - | 2.49 | 169.01 | C_7_H_6_O_5_ | Gallic acid |
| 20 | - | 2.71 | 329.09 | C_13_H_16_O_7_ | Helicid |
| 21 | + | 2.86 | 227.1 | C_10_H_14_N_2_O_4_ | S-(-)-Carbidopa |
| 22 | + | 2.89 | 166.09 | C_9_H_11_NO_2_ | L-Phenylalanine |
| 23 | + | 2.95 | 220.12 | C_9_H_17_NO_5_._0_._5_Ca | Calcium pantothenate |
| 24 | - | 3.26 | 197.05 | C_9_H_10_O_5_ | Danshensu |
| 25 | + | 3.63 | 207.07 | C_11_H_10_O_4_ | Scoparone |
| 26 | + | 3.63 | 225.08 | C_11_H_12_O_5_ | Sinapic acid |
| 27 | - | 4.07 | 153.02 | C_7_H_6_O_4_ | Protocatechuic acid |
| 28 | - | 4.14 | 153.06 | C_8_H_10_O_3_ | 3,4-Dihydroxyphenylethanol |
| 29 | - | 4.21 | 419.12 | C_16_H_22_O_10_ | Swertiamarin |
| 30 | + | 4.22 | 438.16 | C_17_H_24_O_12_ | Sesamoside |
| 31 | + | 4.25 | 272.13 | C_16_H_17_NO_3_ | Higenamine |
| 32 | + | 4.25 | 478.16 | C_19_H_24_O_13_ | Parishin E |
| 33 | - | 4.34 | 183.03 | C_8_H_8_O_5_ | Methyl gallate |
| 34 | + | 4.39 | 360.17 | C_16_H_22_O_8_ | Coniferin |
| 35 | + | 4.45 | 450.16 | C_18_H_24_O_12_ | Asperulosidic acid |
| 36 | + | 4.58 | 205.1 | C_11_H_12_N_2_O_2_ | L-Tryptophan |
| 37 | + | 4.88 | 133.06 | C_9_H_8_O | Cinnamaldehyde |
| 38 | + | 4.88 | 390.18 | C_17_H_24_O_9_ | Eleutheroside B/Syringin |
| 39 | - | 5.23 | 353.09 | C_16_H_18_O_9_ | Chlorogenic acid |
| 40 | + | 5.24 | 163.04 | C_9_H_6_O_3_ | 7-Hydroxycoumarin |
| 41 | + | 5.24 | 355.1 | C_16_H_18_O_9_ | Neochlorogenic acid |
| 42 | + | 5.41 | 328.15 | C_19_H_21_NO_4_ | (-)-Sinoacutine |
| 43 | - | 5.66 | 523.17 | C_21_H_32_O_15_ | Melittoside |
| 44 | - | 5.66 | 137.02 | C_7_H_6_O_3_ | Salicylic acid |
| 45 | - | 5.78 | 281.14 | C_15_H_22_O_5_ | Octyl gallate |
| 46 | + | 5.83 | 286.14 | C_17_H_19_NO_3_ | Piperine |
| 47 | + | 6.00 | 209.04 | C_10_H_8_O_5_ | Fraxetin |
| 48 | + | 6.17 | 439.16 | C_21_H_26_O_10_ | Sec-O-Glucosylhamaudol |
| 49 | + | 6.40 | 197.08 | C_10_H_12_O_4_ | Cantharidin |
| 50 | + | 6.60 | 595.17 | C_27_H_30_O_15_ | 4'-O-Glucosylvitexin |
| 51 | - | 6.75 | 179.03 | C_9_H_8_O_4_ | Caffeic acid |
| 52 | + | 6.89 | 746.25 | C_32_H_40_O_19_ | Parishin C |
| 53 | - | 6.94 | 167.03 | C_8_H_8_O_4_ | 4-Methoxysalicylic acid |
| 54 | + | 7.15 | 342.17 | C_20_H_24_NO_4_ | (+)-Magnoflorine |
| 55 | + | 7.62 | 245.12 | C_15_H_16_O_3_ | Linderalactone |
| 56 | + | 7.86 | 303.05 | C_15_H_10_O_7_ | Quercetin |
| 57 | + | 7.89 | 177.05 | C_10_H_8_O_3_ | 4-Methylumbelliferone |
| 58 | + | 7.89 | 565.16 | C_26_H_28_O_14_ | Isoschaftoside |
| 59 | + | 7.89 | 330.17 | C_19_H_23_NO_4_ | Sinomenine |
| 60 | - | 8.04 | 341.12 | C_15_H_20_O_6_ | Rosin |
| 61 | + | 8.39 | 479.08 | C_21_H_18_O_13_ | Quercetin 3-O-beta-D-Glucuronide |
| 62 | - | 9.04 | 151.04 | C_8_H_8_O_3_ | (R)-Mandelic acid |
| 63 | - | 9.24 | 163.04 | C_9_H_8_O_3_ | p-Hydroxy-cinnamic acid |
| 64 | + | 9.46 | 191.11 | C_12_H_14_O_2_ | 3-n-Butylphathlide |
| 65 | + | 9.46 | 217.05 | C_12_H_8_O_4_ | 8-Methoxypsoralen |
| 66 | + | 9.49 | 611.16 | C_27_H_30_O_16_ | Rutin |
| 67 | + | 9.66 | 549.16 | C_26_H_28_O_13_ | Puerarin apioside |
| 68 | + | 9.83 | 231.14 | C_15_H_18_O_2_ | Atractylenolide I |
| 69 | + | 9.86 | 538.23 | C_26_H_32_O_11_ | Pinoresinol 4-O-glucoside |
| 70 | + | 10.10 | 447.13 | C_22_H_22_O_10_ | Calycosin-7-O-beta-D-glucoside |
| 71 | - | 10.18 | 193.05 | C_10_H_10_O_4_ | Ferulic acid |
| 72 | + | 10.30 | 463.09 | C_21_H_18_O_12_ | Luteolin 7-glucuronide |
| 73 | + | 10.36 | 223.06 | C_11_H_10_O_5_ | Fraxinol |
| 74 | - | 10.38 | 449.11 | C_21_H_22_O_11_ | Astilbin |
| 75 | - | 10.38 | 623.2 | C_29_H_36_O_15_ | Forsythoside I |
| 76 | + | 10.53 | 271.06 | C_15_H_10_O_5_ | Genistein |
| 77 | + | 10.53 | 433.11 | C_21_H_20_O_10_ | Genistin |
| 78 | + | 10.83 | 369.18 | C_21_H_24_N_2_O_4_ | Mitraphylline |
| 79 | + | 10.97 | 449.11 | C_21_H_20_O_11_ | Cynaroside |
| 80 | + | 11.07 | 324.12 | C_19_H_18_NO_4_ | Demethyleneberberine |
| 81 | + | 11.34 | 153.13 | C_10_H_16_O | Camphor |
| 82 | + | 11.34 | 233.12 | C_14_H_16_O_3_ | Fraxinellone |
| 83 | + | 11.34 | 579.17 | C_27_H_30_O_14_ | Rhoifolin |
| 84 | + | 11.44 | 352.12 | C_20_H_17_NO_5_ | Oxyberberine |
| 85 | + | 11.44 | 356.19 | C_21_H_25_NO_4_ | Tetrahydropalmatine |
| 86 | + | 11.58 | 368.15 | C_21_H_21_NO_5_ | (+)-corynoline |
| 87 | + | 11.88 | 209.12 | C_12_H_16_O_3_ | beta-Asarone |
| 88 | + | 11.98 | 609.18 | C_28_H_32_O_15_ | Diosmin |
| 89 | + | 12.08 | 209.08 | C_11_H_12_O_4_ | Methyl 4-hydroxy-3-methoxycinnamate |
| 90 | + | 12.08 | 357.13 | C_20_H_20_O_6_ | Sauchinone |
| 91 | + | 12.22 | 261.11 | C_15_H_16_O_4_ | Linderane |
| 92 | + | 12.32 | 282.17 | C_15_H_23_NO_4_ | Actidione |
| 93 | + | 12.52 | 317.21 | C_20_H_28_O_3_ | Cafestol |
| 94 | - | 12.57 | 549.16 | C_26_H_30_O_13_ | Liguiritigenin-7-O-beta-D-apiosyl-4'-O-beta-D-glucoside |
| 95 | - | 12.64 | 515.12 | C_25_H_24_O_12_ | Isochlorogenic acid B |
| 96 | + | 12.79 | 233.15 | C_15_H_20_O_2_ | Atractylenolide II |
| 97 | - | 12.91 | 187.1 | C_9_H_16_O_4_ | Azelaic acid |
| 98 | - | 13.08 | 359.08 | C_18_H_16_O_8_ | Rosmarinic acid |
| 99 | + | 13.64 | 370.2 | C_22_H_27_NO_4_ | Corydaline |
| 100 | + | 13.81 | 383.2 | C_22_H_26_N_2_O_4_ | Corynoxeine |
| 101 | - | 13.89 | 845.49 | C_42_H_72_O_14_ | Ginsenoside Rg1 |
| 102 | - | 13.96 | 991.55 | C_48_H_82_O_18_ | Ginsenoside Re |
| 103 | + | 14.08 | 207.1 | C_12_H_14_O_3_ | Ethyl 4-methoxycinnamate |
| 104 | + | 14.56 | 447.09 | C_21_H_18_O_11_ | Baicalin |
| 105 | + | 14.69 | 363.18 | C_20_H_26_O_6_ | Ponicidin/Rubescensin B |
| 106 | + | 14.80 | 385.21 | C_22_H_28_N_2_O_4_ | Corynoxine |
| 107 | + | 15.24 | 235.17 | C_15_H_22_O_2_ | Artemisinic acid |
| 108 | + | 15.28 | 352.15 | C_21_H_22_NO_4_ | Palmatine |
| 109 | + | 15.41 | 552.24 | C_27_H_34_O_11_ | Styraxlignolide F |
| 110 | + | 15.55 | 301.07 | C_16_H_12_O_6_ | Hispidulin |
| 111 | + | 15.72 | 336.12 | C_20_H_18_NO_4_ | Berberine |
| 112 | + | 15.79 | 247.13 | C_15_H_18_O_3_ | Arglabin |
| 113 | + | 15.86 | 593.19 | C_28_H_32_O_14_ | Linarin |
| 114 | + | 16.54 | 366.17 | C_22_H_24_NO_4_ | Dehydrocorydaline |
| 115 | + | 16.78 | 251.16 | C_15_H_22_O_3_ | Nardosinone |
| 116 | - | 16.91 | 407.13 | C_20_H_24_O_9_ | Tinnevellin glucoside |
| 117 | + | 16.92 | 247.1 | C_14_H_14_O_4_ | Decursinol |
| 118 | - | 17.42 | 431.1 | C_21_H_20_O_10_ | Apigenin-7-O-beta-D-glucoside |
| 119 | + | 17.68 | 461.11 | C_22_H_20_O_11_ | Wogonoside |
| 120 | + | 18.13 | 355.2 | C_21_H_26_N_2_O_3_ | Vincamine |
| 121 | + | 18.23 | 309.17 | C_17_H_24_O_5_ | 1-O-Acetylbritannilactone |
| 122 | + | 18.44 | 189.09 | C_12_H_12_O_2_ | 3-Butylidenephthalide |
| 123 | + | 18.78 | 249.15 | C_15_H_20_O_3_ | Atractylenolide III |
| 124 | + | 19.40 | 367.2 | C_22_H_26_N_2_O_3_ | Hirsuteine |
| 125 | + | 19.68 | 425.38 | C_30_H_48_O | Lupenone |
| 126 | + | 20.30 | 369.22 | C_22_H_28_N_2_O_3_ | Hirsutine |
| 127 | - | 20.63 | 829.5 | C_42_H_72_O_13_ | Ginsenoside Rg2 |
| 128 | - | 21.01 | 955.49 | C_48_H_76_O_19_ | GinsenosideRo |
| 129 | - | 21.14 | 263.13 | C_15_H_20_O_4_ | Abscisic acid |
| 130 | - | 22.93 | 821.4 | C_42_H_62_O_16_ | Glycyrrhizic acid |
| 131 | - | 23.62 | 515.19 | C_26_H_30_O_8_ | Limonin |
| 132 | + | 25.00 | 193.12 | C_12_H_16_O_2_ | Senkyunolide A |
| 133 | - | 25.05 | 597.27 | C_32_H_40_O_8_ | Handelin |
