# Supplementary material for: Qu-Du-San-Jie decoction induces growth inhibition and vascular normalization in NF2-associated vestibular schwannoma
Source: Front Pharmacol. 2022 Aug 19;13:941854. doi: 10.3389/fphar.2022.941854 (PMC9437245; doi:10.3389/fphar.2022.941854)
Supplement: Supplementary file 3 [file Table3.docx]

Table S3. Top 15 key targets in the PPI network of QDSJ decoction and NF2-associated VS

| **Gene** | **Degree** | **Protein** | **Pathway** |
| --- | --- | --- | --- |
| EGFR | 42 | EGFR | MAPK signaling pathway/ErbB signaling pathway/Ras signaling pathway/Rap1 signaling pathway/HIF-1 signaling pathway/PI3K-Akt signaling pathway/Tumor Angiogenesis |
| PTPRC | 37 | CD45 | Cell adhesion molecules/T cell receptor signaling pathway |
| GRB2 | 36 | GRB2 | MAPK signaling pathway/ErbB signaling pathway/Ras signaling pathway/Rap1 signaling pathway/HIF-1 signaling pathway/PI3K-Akt signaling pathway |
| ERBB2 | 35 | HER2 | MAPK signaling pathway/ErbB signaling pathway/PI3K-Akt signaling pathway/HIF-1 signaling pathway |
| FGF2 | 31 | FGF2 | MAPK signaling pathway/Ras signaling pathway/PI3K-Akt signaling pathway/Tumor Angiogenesis |
| CCND1 | 31 | cyclin D1 | Cell cycle/PI3K-Akt signaling /FoxO signaling pathway/p53 signaling pathway |
| PPARG | 30 | PPARγ | PPAR signaling pathway/AMPK signaling pathway |
| HPGDS | 29 | HPGDS | Glutathione metabolism/Drug metabolism - cytochrome P450 |
| KDR | 28 | VEGFR2 | MAPK signaling pathway/Ras signaling pathway/PI3K-Akt signaling pathway/Tumor Angiogenesis |
| STAT1 | 22 | STAT1 | Necroptosis/Chemokine signaling pathway/JAK-STAT signaling pathway |
| LYN | 21 | LYN | Chemokine signaling pathway/NF-kappa B signaling pathway |
| SYK | 20 | SYK | PI3K-Akt signaling /NF-kappa B signaling pathway |
| MET | 16 | MET | MAPK signaling pathway/ErbB signaling pathway/PI3K-Akt signaling/Fibrosis |
| FGF1 | 12 | FGF1 | MAPK signaling pathway/Ras signaling pathway/PI3K-Akt signaling pathway/Tumor Angiogenesis |
| PDGFRA | 11 | PDGFRA | MAPK signaling pathway/Ras signaling pathway/PI3K-Akt signaling pathway/Tumor Angiogenesis |
