# Supplementary figures and images for: Qu-Du-San-Jie decoction induces growth inhibition and vascular normalization in NF2-associated vestibular schwannoma
Source: Front Pharmacol. 2022 Aug 19;13:941854. doi: 10.3389/fphar.2022.941854 (PMC9437245; doi:10.3389/fphar.2022.941854)

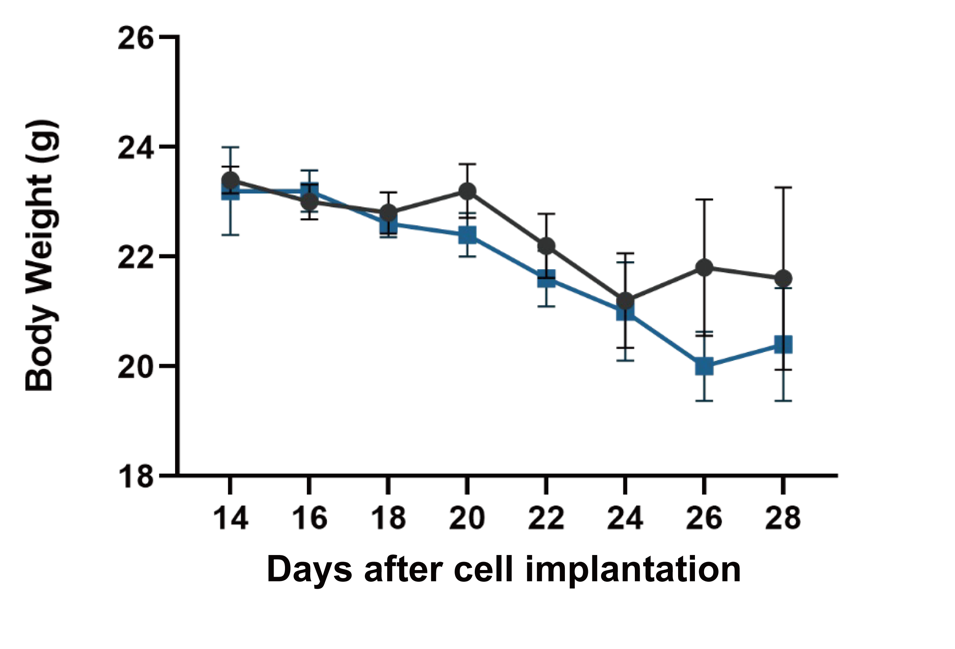


**Figure S2**. Body weight of NF2-associated VS xenograft mice after QDSJ decoction treatment.

Supplement: Supplementary file 4 [file DataSheet2.docx]
