# Supplementary material for: Qu-Du-San-Jie decoction induces growth inhibition and vascular normalization in NF2-associated vestibular schwannoma
Source: Front Pharmacol. 2022 Aug 19;13:941854. doi: 10.3389/fphar.2022.941854 (PMC9437245; doi:10.3389/fphar.2022.941854)
Supplement: Supplementary file 5 [file DataSheet1.docx]

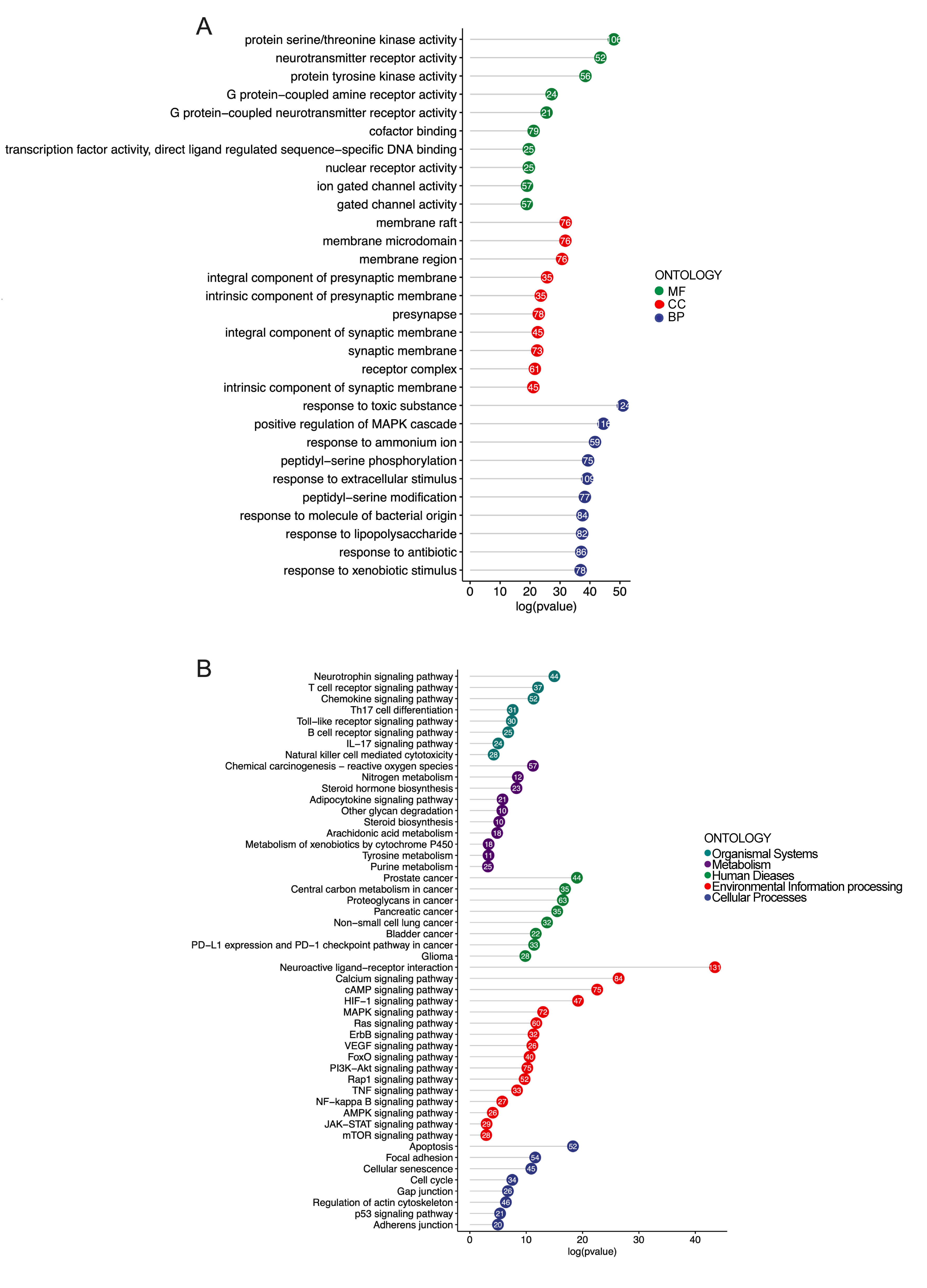


**Figure S1.** Enrichment analysis for candidate targets of QDSJ decoction. (A) Go terms of the candidate targets of QDSJ. The top 10 GO functional categories were selected. (B) KEGG pathway enrichment of the candidate targets of QDSJ.
